# Genomic characterization of *Lacticaseibacillus paracasei* strains from Greek fermented olives reveals common and unique characteristics and distinct clades

**DOI:** 10.1128/aem.00515-26

**Published:** 2026-03-30

**Authors:** Kyriaki Feidaki, Yuchen Yan, Bowen Yang, Anagnostis Argiriou, Panayiotis Panas, Yiannis Kourkoutas, Robert Hutkins, Yanbin Yin

**Affiliations:** 1 Department of Food Science and Nutrition, University of the Aegean 551823, Myrina, Lemnos, Greece; 2 Nebraska Food for Health Center, Department of Food Science and Technology, University of Nebraska-Lincoln 14719, Lincoln, Nebraska, USA; 3 Institute of Applied Biosciences, Centre for Research and Technology Hellas 419215, Thessaloniki, Greece; 4 QLC 142653 , Patras, Greece; 5 Department of Molecular Biology & Genetics, Democritus University of Thrace, Alexandroupolis, Greece; Universita degli Studi di Napoli Federico II, Portici, Italy

**Keywords:** pangenome, Greek fermented food, prebiotics, CAZymes, *Lacticaseibacillus paracasei*

## Abstract

In this study, we isolated and sequenced the genomes of three lactic acid bacteria derived from Greek fermented table olives. The isolates, OLXI-1, OLXI-6, and OLXI-9, all belonged to the species *Lacticaseibacillus paracasei,* and a phylogenomic analysis revealed they clustered into three distinct clades. A pangenome analysis was performed with 220 public *Lc. paracasei* genomes and the three olive isolates. The source (dairy, non-dairy) and the geographical information (Asia, Europe, North America, South America, Africa) of each strain were mapped onto the pan-genome and the phylogeny to inform the possible inter-environmental transmission of *Lc. paracasei*. Based on the carbohydrate-active enzyme (CAZyme) annotation of the *Lc. paracasei* genomes, we identified unique gene clusters and CAZymes relevant to their physiological role in fermented foods, as well as traits that may contribute to health benefits. We also compared *in silico* results with functional *in vitro* experiments to assess growth on different prebiotic substrates (fructooligosaccharides [FOS], XOS, galactooligosaccharides [GOS], and inulin), growth in olive pomace, and in the production of inhibitory substances. Notably, consistent with the observed presence of the relevant CAZymes, the olive-derived strain, OLXI-9, grew best on the two fructans, inulin and FOS, with cell densities even higher than glucose.

**IMPORTANCE:**

Fermentation has long been used to preserve and enhance the shelf-life, flavor, texture, and functional properties of food. Table olives, in particular, are well known for their sensory and nutritional properties, and more recently, the microbiota of fermented olives has been suggested to contribute potential benefits in the gastrointestinal tract. In this study, a strain of *Lacticaseibacillus paracasei*, isolated from Greek fermented table olives, had the genetic and physiological capacity to consume prebiotic substrates. These findings provide a basis for understanding how fermentation-associated microbes grow on prebiotic fibers and potentially contribute to human health.

## INTRODUCTION

Table olives have been produced and consumed in the Mediterranean basin for thousands of years and are now produced and consumed worldwide (1). During 2023–2024, the production of table olives reached nearly 3 million tons, with Egypt, Turkey, Spain, Algeria, and Greece among the main producers (2). The popularity of table olives is due, in part, to their nutritional attributes that include a wide variety of phenol and polyphenol compounds (3, 4). Based on the way they are produced, table olives can be generally divided into Spanish-style, Greek-style, and California-style (1). Of these, only the Spanish- and Greek-style olives are fermented. In addition, Greek-style or naturally black, ripe-style olives are not treated with lye prior to fermentation in brine (5). Moreover, lower brine concentrations, 6% to 8% NaCl for 8–12 months (6), may contribute to a more diverse microbiota, with lactic acid bacteria (LAB) from the genus *Lactiplantibacillus* dominating spontaneous fermentations, as well as the olive surface (7). Collectively*, Lactiplantibacillus pentosus*, *Lactiplantibacillus plantarum*, and *Lacticaseibacillus paracasei,* together with diverse species of yeasts, are among the main microorganisms that ferment table olives (8).

*Lacticaseibacillus paracasei* species is divided into two subspecies, *Lc. paracasei* subsp. *paracasei* and *Lc. paracasei* subsp. *tolerans*. They are facultative anaerobes that have a broader carbohydrate utilization capacity compared to other LAB and as a result have adapted to several niches, from foods to host-associated microenvironments (9–11). In particular, it has been reported that *Lc. paracasei* strains can consume several prebiotic carbohydrates, including inulins, fructooligosaccharides (FOS), and galactooligosaccharides (GOS) (9–11) that may provide a competitive advantage when these carbohydrates are present. Whole-genome sequencing and comparative genomic analysis have previously been used for taxonomic assignment of *Lacticaseibacillus* and to screen for metabolic activities, potential virulence factors, and to assess for probiotic traits (12–15). Indeed, as noted recently, strains of *Lc. paracasei* have a long history of use as a probiotic (13).

In this study, we describe the genomic and phenotypic properties of three strains of *Lc. paracasei*, OLXI-1, OLXI-9, and OLXI-6, that were isolated from Greek table olives. Preliminary evaluation, based on a series of established *in vitro* tests, including tolerance to bile salts and acidic pH, as well as susceptibility to common antibiotics. These findings suggested that the three isolates possessed traits commonly associated with probiotic potential. Notably, previous work from our group has shown that other olive-derived lactic acid bacteria can exert beneficial metabolic effects *in vivo*, raising the possibility that table olives can serve as a reservoir of functionally relevant strains (16). Therefore, our goals in the present study were to use comparative genomics to relate these *Lc. paracasei* isolates to other publicly available genomes, to examine the broader genome diversity of this species, and to characterize the probiotic and physiological attributes of the strain OLXI-9.

## MATERIALS AND METHODS

### Bacterial strains

*Lc. paracasei* OLXI-1, OLXI-6, and OLXI-9 were isolated from Greek fermented olives as previously described (16). Briefly, samples were grown in MRS broth, and portions were plated onto acidified MRS agar (Condalab, Madrid, Spain). Isolates with typical morphology on agar plates were selected and used for phenotypic identification and then identified by 16S rRNA gene sequencing (16). Strains were subsequently stored at −80°C in MRS broth containing glycerol (25% vol/vol) and were sub-cultured routinely in MRS broth (Oxoid, Milan, Italy) at 37°C for 24 h prior to their use.

### Whole-genome sequencing and *de novo* assembly of *Lc. paracasei* OLXI-1, OLXI-6, and OXLI-9

*Lc. paracasei* strains were cultured overnight in MRS broth at 37°C for 24 h. From each bacterial culture, 600 µL was centrifuged (11,000 × *g* for 3 min), supernatant was removed, and then the cells were resuspended in 750 µL lysis buffer from the DNA Extraction ZymoBIOMICS DNA Miniprep Kit (Zymo Research, Orange, CA, USA). Genomic DNA was extracted using the ZymoBIOMICS DNA Miniprep DNA Extraction Kit, according to the manufacturer’s instructions, in a final elution volume of 50 μL. The DNA concentration for each sample was measured using the Qubit dsDNA BR Assay Kit (Life Technologies, Carlsbad, CA). Genome sequencing of the three isolates was performed using the paired-end sequencing technology with an Illumina MiSeq sequencer and Nextera XT library kit (Illumina, US). The quality of the reads was determined using FASTQC, and *de novo* assembly of the raw reads was performed using the SPAdes *De Novo* Assembler with a stringent parameter to minimize mismatches (17). Assembly metrics were calculated with CheckM (18).

### Genome annotation

Gene annotation was performed using Prokka with standard parameters (19). Prophage regions were detected using VIBRANT (20). Polyphenol utilization genes were identified using dbPUP (21). WGS-based antimicrobial susceptibility was performed using ResFinder 4.1 (22). CRISPRs and Cas were predicted using CRISPRCasFinder implemented in AcrFinder (23). Bacteriocins were predicted with BAGEL4 (24).

### Pangenome analysis

A pangenomic analysis was performed by combining 220 public genomes of *Lc. paracasei*, four genomes of *L. casei* as outgroup, and the three genomes of our *Lc. paracasei* isolates using Anvi’o v7 (25–27).

### Phylogenomic analysis and visualizations

We used the IQ-Tree tool with default parameters to build the phylogeny (28) using the *concatenated_alignment.fa* file generated by Anvi’o v7. This file comprises concatenated multiple sequence alignments of single-copy core genes identified across the genomes of interest, providing a robust data set for high-resolution phylogenomic tree construction. We visualized the tree using the “Interactive Tree of Life” tool (29).

### Carbohydrate-active enzyme analysis

Identification of genes related to carbohydrate-active enzyme (CAZyme) families, including glycoside hydrolase (GH), glycosyl transferase (GT), polysaccharide lyase (PL), carbohydrate esterase (CE), carbohydrate binding module (CBM), and auxiliary activity (AA), was performed using the run_dbcan program of dbCAN2 with default settings and parameters (30).

### Carbohydrate utilization

The biochemical profile, based on carbohydrate utilization, of *Lc. paracasei* OLXI-9 was determined using the API 50 CHL (bioMérieux, Marcy-l’Étoile, France) arrays, according to the manufacturer’s protocol.

### Growth on prebiotics and olive pomace

Growth of OLXI-9 on prebiotics was performed in 96-well microtiter plates (Sigma SIAL0596, MO, USA) using an automated plate incubator and OD reader (Tecan). Briefly, overnight cultures were centrifuged at 3,000 × *g* for 5 min, and pellets were washed twice and resuspended in basal MRS (bMRS; no added carbohydrate). Suspensions were then inoculated (1%), in triplicate, into bMRS containing sterile-filtered XOS (Prenexus), FOS (Orafti), GOS (Friesland), or inulin (Orafti) at 0.5% final concentration. All prebiotics were more than 90% pure. Plates were incubated at 37°C for 24 h, and growth was measured at 600 nm every 30 min. Positive controls contained glucose, and negative controls contained bMRS without added carbohydrate (31).

To assess fermentation of olive pomace substrates following previous papers (32–34), *Lc. paracasei* OLXI-9 was inoculated in basal bMRS containing 1% or 2% Olive Powder (OlioCru-PreBio Olive Powder, Trento, Italy). The Olive Powder contained 21.6% sugars. Cultures were incubated at 37°C for 24 h, and fermentation was based on changes in pH.

### Formation of inhibitory compounds

Agar well diffusion assays were used to determine if OLXI-9 produced bacteriocins or other inhibitory substances. Two conditions were tested separately: (i) live OLXI-9 cells, to assess whether direct cell-associated activity inhibits indicator strains, and (ii) cell-free filtrates (CFF) obtained from OLXI-9 overnight cultures, to evaluate whether secreted metabolites or bacteriocins alone were inhibitory. As a negative control, MRS broth was included to confirm that any observed inhibition was not due to the medium. Indicator organisms included four *Listeria monocytogenes* strains and single strains of *Enterococcus lactis* and *Enterococcus xiangfangensis*. Overnight cultures of each indicator strain (grown in LB at 37°C for 12–18 h) were adjusted to ~10⁸ CFU/mL, and 1-mL aliquots were spread onto BHI or LB agar plates. Five 6-mm wells were cut into the agar and filled in duplicate with 100 µL of each test condition (live OLXI-9, CFF) and MRS control. Plates were held at 4°C for 2 h to allow diffusion, then incubated at 37°C for 24 h before examining zones of inhibition.

### Evaluation of β-glucosidase activity

β-glucuronidase activity in OLXI-9 was assessed using the fluorogenic substrate 4-methylumbelliferyl-β-D-glucuronide as described previously (10). *Lc. rhamnosus* OLXAL-2, previously shown to exhibit α-glucosidase inhibitory activity (35), was included as a positive control. Protein concentrations in cell fractions were determined using the Lowry method (36).

## RESULTS

### Genome features

Whole-genome sequencing, *de novo* assembly, and genome annotation were performed to investigate the genomic features of *Lc. paracasei* strains OLXI-1, OLXI-6, and OLXI-9. All three genomes were within the expected size and GC content ranges reported for this species, confirming their taxonomic placement and genomic stability (12–15). The number of predicted genes and coding sequences (CDSs) identified in each strain also aligned with previously characterized *Lacticaseibacillus* genomes, while some variation was observed in the numbers of rRNAs and tRNAs (Table 1). These differences may reflect strain-specific genomic adaptations or incomplete annotations commonly encountered in draft genomes. Notably, OLXI-1 exhibited a higher number of rRNA genes compared to OLXI-9 and OLXI-6, which could suggest increased transcriptional capacity or differing genome organization.

**TABLE 1:** Genome characteristics of OLXI-1, OLXI-9, and OLXI-6

Sample ID	OLXI-9	OLXI-1	OLXI-6
Source	Table olives	Table olives	Table olives
Method	Illumina/MinION	Illumina	Illumina
Number of contigs	54	80	83
Total read counts	2,864,638	4,750,379	1,081,182
Total read length (mbp)	1,718	2,850	649
Contamination %	0.00	0.00	0.00
N50 (bp)	118,994	128,535	97,580
Completeness %	99.5	99.5	99.5
Genome length (bp)	3,109,455	3,091,733	3,110,818
GC content %	46	46	45
Predicted genes	2,984	3,015	3,043
CDSs	2,969	2,985	2,997
rRNAs	5	41	6
tRNAs	55	57	53
tmRNAs	1	1	1
misc_RNAs	31	31	31
CRISPR arrays	1	1	1
Cas proteins	3	1	1
Prophages	4	7	7

An abundance of prophages in the genomes of *the Lactobacillaceae* has been previously reported (37). Accordingly*, Lc. paracasei* OLXI-9, OLXI-1, and OLXI-6 contained 4, 7, and 7 prophage regions, respectively. Each of these isolates also harbored 1 CRISPR array (Table 1) and genes encoding for 3, 1, and 1 Cas proteins, respectively, which may contribute to phage resistance. These strains did not harbor antibiotic resistance genes or virulence factors.

### Phylogeny reveals three clades of *Lc. paracasei*

**Fig 1:**
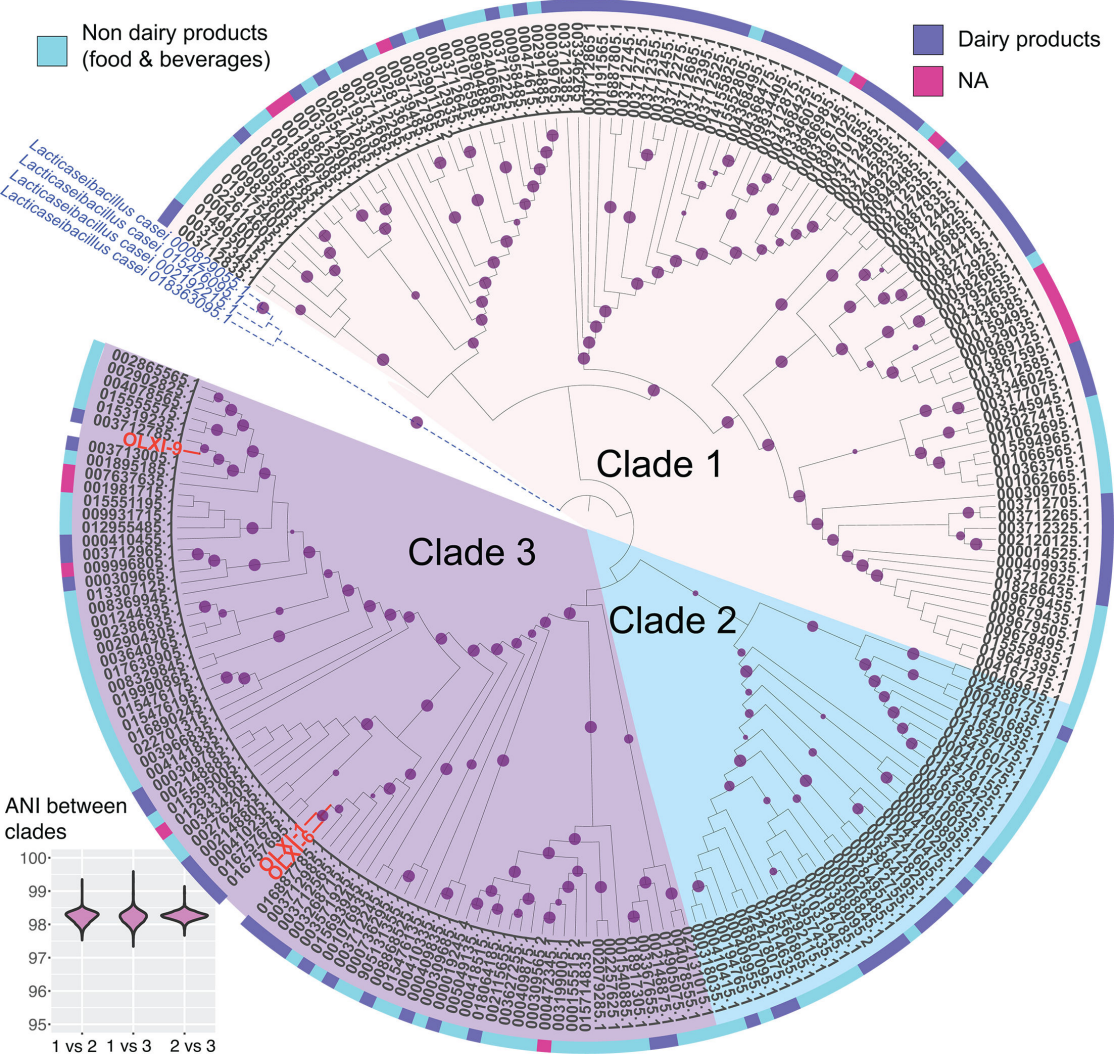
:Phylogeny tree of *Lc. paracasei* genomes. Two hundred twenty genomes are from NCBI, plus three of our isolates and four *Lc. casei* genomes as an out-group. Leaves are GenBank assembly IDs (the prefix “GCF_” is removed for better presentation). Bootstrap values are featured in dark purple dots in the range 70–100, and our three isolates are marked in red fonts. The genomes are classified into three major clades that are accordingly related to the isolation source of the strains, dairy or non-dairy food and beverages. Clade 1 (background color light beige) appears to consist of genomes derived mainly from dairy products, whereas clade 2 (color light green) and clade 3 (color light purple) genomes were isolated mainly from non-dairy food and beverages. Our isolates were clustered together into clade 3, but in two different sub-clades. Isolates OLXI-1, OLXI-9, and OLXI-6 have the closest evolutionary relationship to genomes isolated from dairy products. Average nucleotide identity (ANI) was calculated for genomes in each clade pair, and the boxplot is shown in the bottom left corner.

The classification of 223 *Lc. paracasei* genomes, enabled by phylogeny with 4 *Lc. casei* genomes as the out-group, showed three major clades (Fig. 1). Clade 1 contains 106 genomes, including 59 from dairy products, 36 from non-dairy food and beverages, and 11 of unknown origin. Clade 2 consists of 36 genomes, with 23 from non-dairy food and beverages and 13 from dairy sources. Finally, clade 3 contains OLXI-1, OLXI-9, and OLXI-6, and 81 public genomes with 53 isolates from non-dairy food and beverages, 23 from dairy products, and 5 from unknown origin.

*Lc. paracasei* has been used as a starter culture for cheese and cultured dairy products and is also found in a variety of naturally fermented products, including fermented dairy and fermented vegetables (38). The phylogeny shows that clade 1 contained strains mainly derived from dairy products, whereas clades 2 and 3 contain strains mainly from non-dairy sources. Interestingly, OLXI-1, OLXI-9, and OLXI-6 were the only ones derived from fermented olives and are closely clustered with strains from dairy sources in clade 3.

To evaluate the taxonomic status of the three clades identified in the phylogenetic tree, we calculated whole-genome average nucleotide identity (ANI) scores with FastANI (27) between clade pairs. The inter-clade scores were higher than ≥95% (Fig. 1), indicating that all the genomes of the three clades belong to the same species.

### Clade 3 phylogeny with metadata and CAZyme heatmap

**Fig 2:**
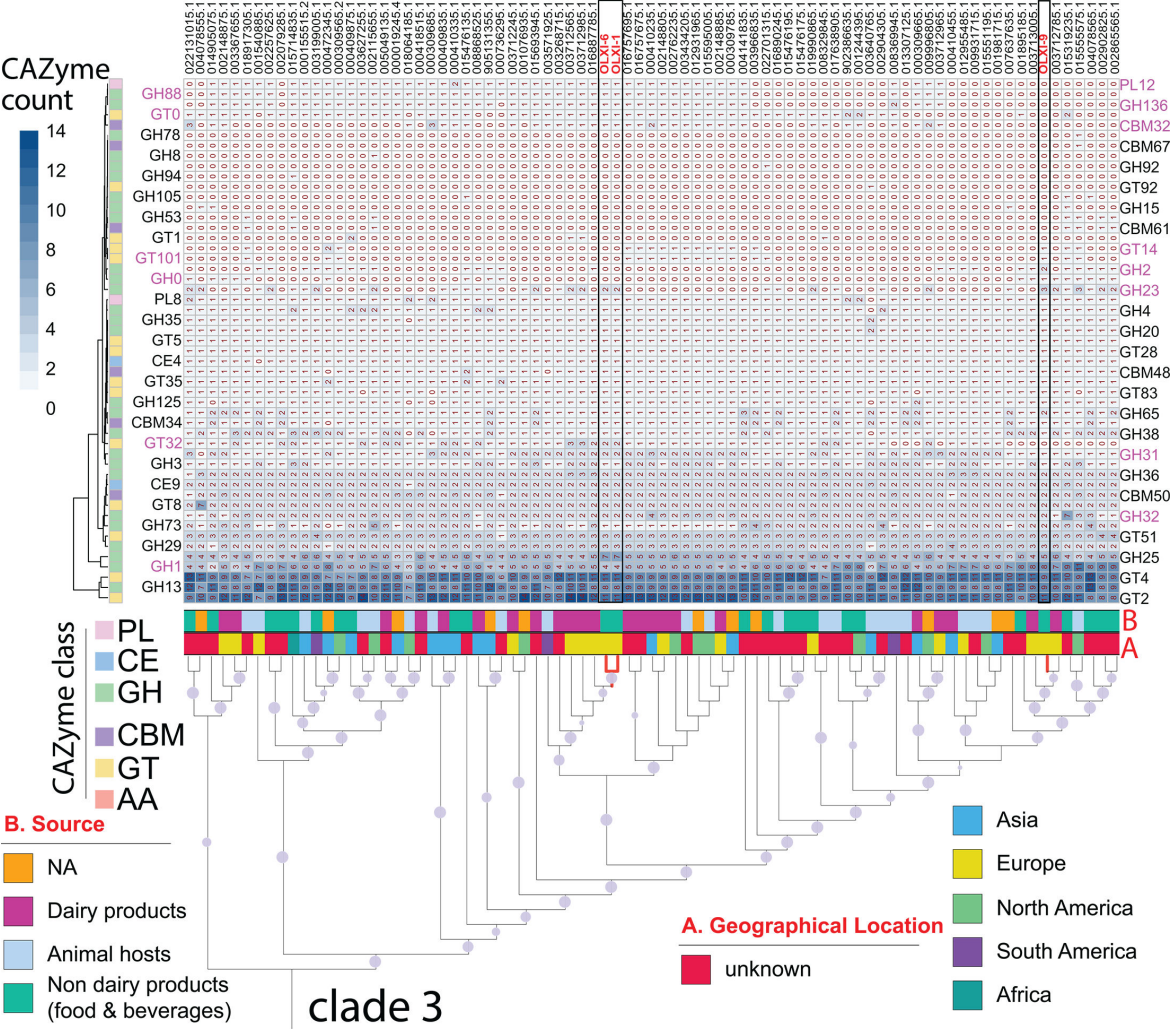
Gene counts of different CAZyme families in genomes of clade 3. This clade contains our three isolates with geographical annotation, source metadata, and CAZyme profile (top heatmap). Leaves in the phylogeny are GenBank assembly IDs (the prefix “GCF_” is removed for better presentation). The tree is a subtree pruned from the complete phylogeny in Fig. 1. The geographical annotation is shown as band A, and the source metadata is shown as band B. In band B, the “animal hosts” is a new category (not in Fig. 1) separated from the “non-dairy products” category. In the CAZyme profile heatmap, each row is a CAZyme family, and each column is a genome. CAZyme family labels (with six colors for six CAZyme classes) are shown on both sides beside their corresponding rows. The pink families are discussed in the main text. Rows are sorted according to the abundance profiles, and the dendrogram is shown on the left representing the CAZyme profile similarity.

Clade 3 was investigated further based on source and geography (Fig. 2). Geographical regions were divided into five groups: Asia, Europe, North America, South America, and unknown. Source annotation was divided into four groups: dairy products, non-dairy food and beverages, animal hosts, and unknown. The animal hosts group included genomes sourced from human gut, human and canine feces, blood, vagina, saliva, and dentine caries. OLXI-1, OLXI-9, and OLXI-6 were clustered with other strains isolated from dairy products or non-dairy food products, and their nearest neighbors were isolates from Europe (Fig. 2). OLXI-9 was clustered in a different sub-clade to OLXI-1 and OLXI-6, whose nearest neighbors were from dairy sources and more specifically *Lc. paracasei* 2306 (GCF_016887785.1) isolated from Parmigiano Reggiano cheese in Italy, FAM8140 (GCF_003712985.1) and FAM19317 (GCF_003712565.1) from Emmental cheese in Switzerland. The closest strains for OLXI-9 are FAM6161 (GCF_003712785.1) and *FAM7821* (GCF_003713005.1) that were both isolated from milk and semi-hard cheese in Switzerland, and *LC-Ikematsu* (GCF_001895185.1) that was isolated from pineapple in Japan.

We further annotated genomes of clade 3 for CAZymes using dbCAN2. CAZymes are divided into six classes that include AA, GT, CBM, GH, CE, and PL. A total of 51 CAZyme families were found in clade 3, including 27 GH, 14 GT, 6 CBM, 2 CE, and 2 PL families (Fig. 2). Isolates OLXI-6 and OLXI-1 are grouped together in the phylogeny. Accordingly, they have a more similar CAZyme profile than OLXI-9. For example, OLXI-6 and OLXI-1 contained families PL12, GH88, GH136, GT0, CBM32, and GT32, which are missing from isolate OLXI-9.

### Pangenome analysis of clade 3

**Fig 3:**
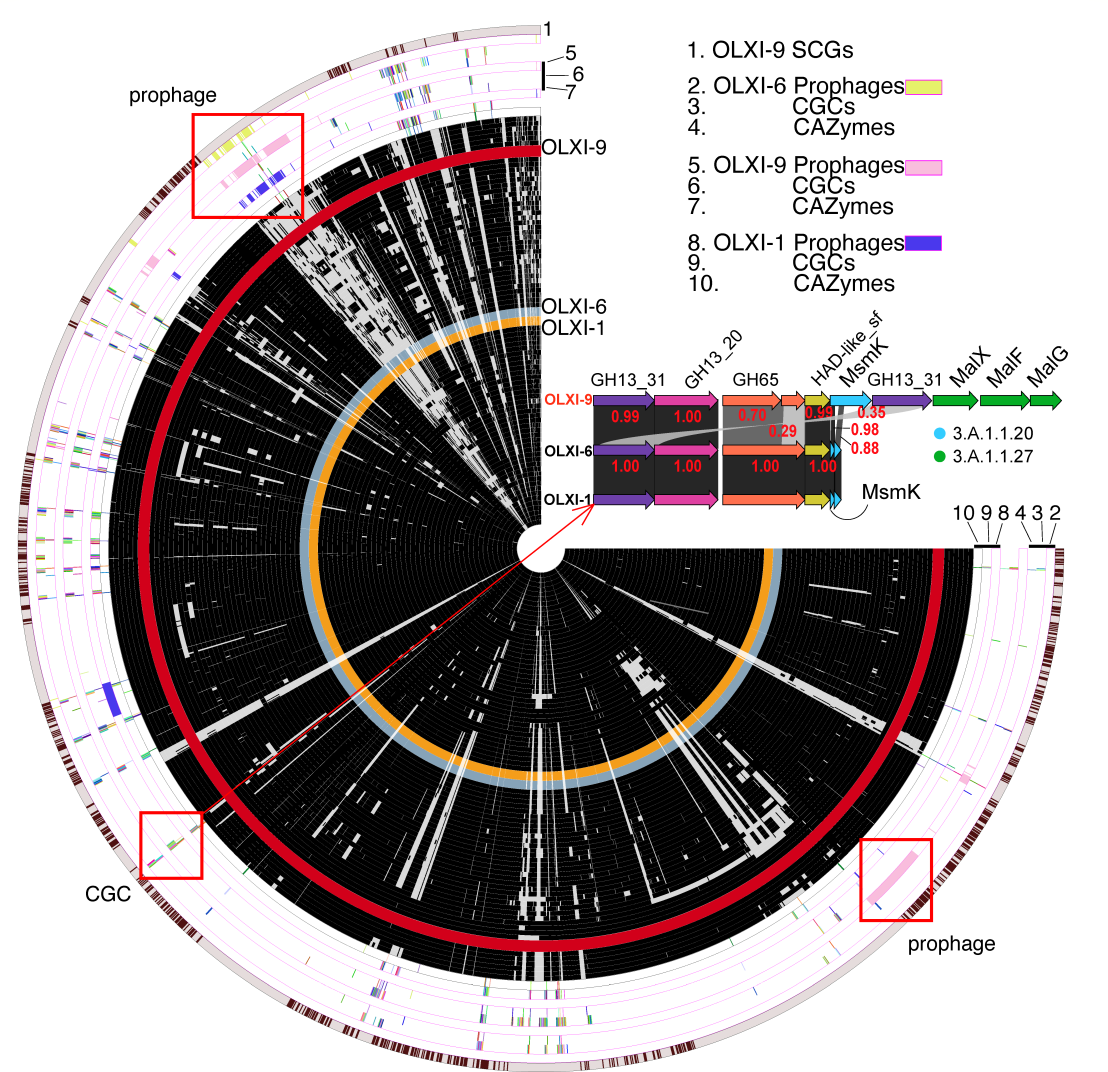
Pan-genome analysis of 81 genomes of clade 3. Anvi’o plot of the pan-genome of 78 *Lc. paracasei* genomes from NCBI, plus our three isolates. From the innermost, each circle represents a genome, and therefore, there are a total of 81 circles. Except for OLXI-1, OLXI-9, and OLXI-6, in all other circles, each tiny black box means the genome contains the gene family, and a gray box means the genome does not contain the gene family. The OLXI-9 circle is all maroon, because only the gene families found in OLXI-9 are plotted here. The gene families are arranged according to their positions in the OLXI-9 genome. Ten rings are used to show the interested genes and genetic elements. SCGs, single copy core genes; CGCs, CAZyme gene clusters; CAZymes, carbohydrate active enzymes. A CGC alignment is shown to depict the similarities among syntenic genes in these CGCs across the three OLXI genomes. Red numbers are sequence identities in fractions.

To compare *Lc. paracasei* genomes and identify core genes, as well as unique genes and functions, a pangenome analysis was performed (26). Accordingly, we plotted the pan-genome of clade 3 (Fig. 3), including all gene families present in OLXI-9 and excluding gene families absent in OLXI-9 (e.g., those only present in OLXI-1 and OLXI-6). All gene families were arranged in the order of their linear positions in the genome of OLXI-9, so that the conservation (presence and absence) of each gene family and each physically linked gene cluster across the 81 *Lc. paracasei* genomes could be observed.

Ten rings are presented in Fig. 3, with the outermost ring being the single copy core genes in OLXI-9 that are also present in all other genomes of clade 3. The other nine rings illustrate the presence/absence of three genetic elements of interest, prophages, carbohydrate gene clusters (CGCs), and CAZymes in the three OLXI genomes. We observed at least four prophages (Table S1) in the OLXI-9 genome, and two of these prophages had large genome sizes (>80 kb), highlighted in pink in ring #5 of Fig. 3. These prophages were also present in OLXI-1 and OLXI-6 but showed various genomic variations. We also highlighted all CAZymes (ring #7 of Fig. 3) and CGCs (ring #6 of Fig. 3) in the pan-genome. CGCs, a more general term of polysaccharide utilization loci, are defined as gene clusters with at least one CAZyme gene, and at least one of the other three types of signature genes: transporter genes, transcription factor genes, and signal transduction proteins (39). Most CGCs were also conserved in the three genomes but had major variations (Table S2).

As an example, we highlighted a CGC (Fig. 3), which is likely involved in starch utilization and exhibits clear differences between OLXI-9 and the other two genomes. In OLXI-9, the CGC contains two GH13_31 genes, two GH65 genes, and four ABC transporter genes. However, OLXI-1 and OLXI-6 only have one GH13_31, one GH65, and two ABC transporter genes. The alignment of the CGC (the two sides of the CGC are conserved in the three genomes) across the three genomes showed that one of the two GH13_31 genes in OLXI-9 has only 35% sequence identity with the single GH13_31 gene in the other two genomes. The two shorter GH65 genes in OLXI-9 correspond to the single longer GH65 gene in the other two genomes. The most significant difference is in the ABC transporters: the two ABC transporters (best hit to MsmK gene in TCDB) are two short fragments of the MsmK gene in OLXI-9, and the three MalX/F/G genes in OLXI-9 are absent in the other two genomes according to a BLASTP search with the threshold sequence identity >30% and coverage >0.8. This can be explained by either a recent horizontal gene transfer into OLXI-9 or a recent loss of these transporter genes in OLXI-1 and OLXI-6. The existence of the complete maltodextrin transporter subunits (MalX/F/G/K) in OLXI-9 suggests a higher efficiency of starch utilization compared to OLXI-1 and OLXI-6, which only have the conserved GH genes and two MsmK fragment genes.

Polyphenol utilization proteins (PUPs) are enzymes that are necessary for the metabolism of polyphenols (21). Searching against the dbPUP database using sequence identity >30% and E-value <1e-10, we found six PUPs in the OLXI-9 genome and four PUPs in the other two genomes (Table 2). Three PUPs are shared by the three genomes: (i) beta-glucosidase (substrate: genistin/glycitin), (ii) beta-glucosidase (substrate: daidzin/genistin), and (iii) amylosucrase (substrate: phloretin). Three PUPs are unique to OLXI-9, including NADH-dependent flavin reductase subunit 1 and subunit 2 (substrate: flavin mononucleotide), and (iv) Enoate reductase (substrate: naringenin). One PUP is absent in OLXI-9 but present in the other two genomes: daidzein reductase (substrate: genistein/daidzein).

**TABLE 2:** Polyphenol utilization proteins in OLXI-1, OLXI-6, and OLXI-9

Gene ID	dbPUP best hit ID	Description	Organism	Substrate	Identity	E-value
OLXI-9						
1018	J9XU85	Beta-glucosidase	*Bifidobacterium lactis*	Genistin/glycitin	30.75	7.99E-68
1053	A0A072N4Q2	Beta-glucosidase	*Bifidobacterium pseudocatenulatum IPLA36007*	Daidzin/genistin	41.97	2.14E-166
1531	Q9ZEU2	Amylosucrase	*Neisseria polysaccharea*	Phloretin	32.22	4.65E-27
2722	Q74HL7	NADH-dependent flavin reductase subunit 1	*Lactobacillus johnsonii*	Flavin mononucleotide	46.51	7.60E-48
2723	Q74HL8	NADH-dependent flavin reductase subunit 2	*Lactobacillus johnsonii*	Flavin mononucleotide	40.98	6.29E-42
548	V9P074	Enoate reductase	*Eubacterium ramulus*	Naringenin	32.12	2.56E-97
OLXI-1						
1088	J9XU85	Beta-glucosidase	*Bifidobacterium lactis*	Genistin/glycitin	31.28	2.61E-57
601	A0A072N4Q2	Beta-glucosidase	*Bifidobacterium pseudocatenulatum IPLA36007*	Daidzin/genistin	41.97	1.33E-166
956	Q9ZEU2	Amylosucrase	*Neisseria polysaccharea*	Phloretin	32.22	4.84E-27
2467	M9NZ71	Daidzein reductase	*Slackia isoflavoniconvertens*	Genistein (daidzein)	31.09	3.48E-31
OLXI-6						
929	J9XU85	Beta-glucosidase	*Bifidobacterium lactis*	Genistin/glycitin	31.28	2.62E-57
334	A0A072N4Q2	Beta-glucosidase	*Bifidobacterium pseudocatenulatum IPLA36007*	Daidzin/genistin	41.97	1.34E-166
709	Q9ZEU2	Amylosucrase	*Neisseria polysaccharea*	Phloretin	32.22	4.86E-27
2468	M9NZ71	Daidzein reductase	*Slackia isoflavoniconvertens*	Genistein (daidzein)	31.09	3.50E-31

### Carbohydrate utilization by *Lc. paracasei* OLXI-9

Among the three isolates, OLXI-9 was subsequently selected for further experimental characterization as a representative strain displaying distinctive genomic features and carbohydrate utilization potential. To assess the diversity of carbohydrates that *Lc. paracasei* OLXI-9 could ferment, API-50CHL test kits were used. The strain fermented 20 out of the 49 carbohydrates included in the panel (Table S3). The specific carbohydrates metabolized by OLXI-9 were generally consistent with *in silico* predictions based on dbCAN analysis. Accordingly, glycoside hydrolase families, such as GH2, which include enzymes involved in the hydrolysis of β-linked sugars, were present and may support the metabolism of complex carbohydrates. While the monosaccharides, galactose, fructose, and mannose do not require glycosidic bond cleavage, their utilization reflects possible cross-feeding and the strain’s broader carbohydrate metabolic capacity, which aligns with its CAZyme profile. Similarly, the presence of GH32 family enzymes, known to include inulinases, corresponds to the observed inulin fermentation *in vitro*.

**Fig 4:**
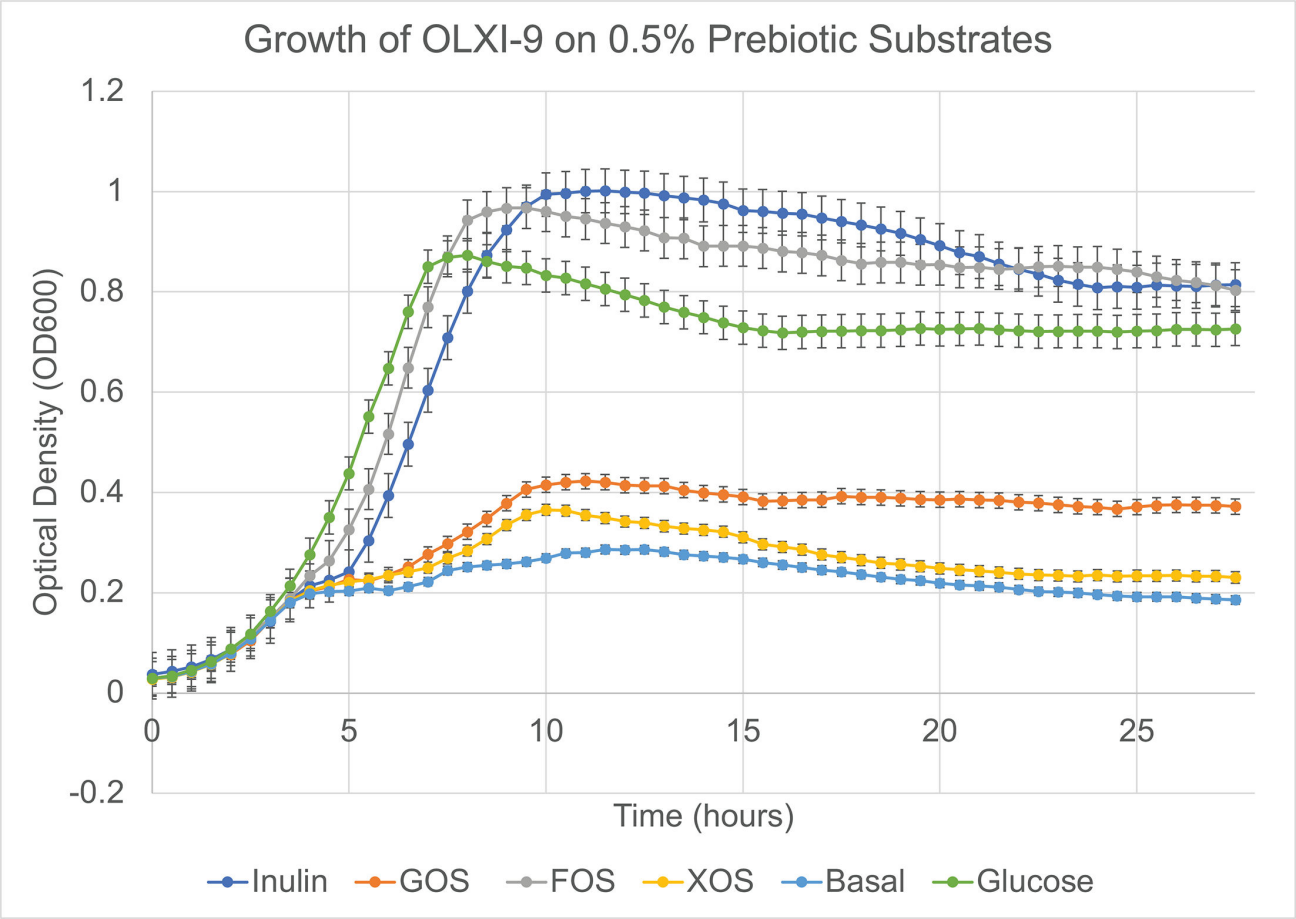
Growth curves of *Lacticaseibacillus paracasei* OLXI-9 on different prebiotic substrates. Optical density at 600 nm (OD600) was measured during growth in MRS supplemented with 0.5% (wt/vol) inulin, FOS, GOS, or XOS. Data represent the mean ± standard deviation of three independent biological replicates, each performed in technical triplicate; error bars indicate SD. OLXI-9 displayed significantly higher growth on inulin and FOS during the exponential phase, consistent with the presence of GH32 fructan-utilization genes, while limited growth on GOS and XOS corresponds with the absence of GH11 and CE genes associated with their metabolism.

In addition to inulin, growth of *Lc. paracasei* OLXI-9 on three other prebiotic substrates, GOS, FOS, and XOS, was also determined (Fig. 4). Interestingly, *Lc. paracasei* OLXI-9 grew best on inulin and FOS, with cell densities higher than glucose. This result, as before, is consistent with the observed presence of the CAZyme families, such as GH32, that were found *in silico*. In contrast, the absence of genes associated with the utilization of GOS and XOS (GH11 and CEs) in this strain was also consistent with its inability to grow on these substrates.

Because *Lc. paracasei* OLXI-9 had been isolated from olive fermentations, we assessed the ability of this strain to ferment carbohydrates in an olive pomace powder. The fermentation was based on the decrease in pH. The experiment, conducted in triplicate, showed a modest decrease in the mean pH, from 6.34 to 5.51.

### Production of inhibitory substances

Genes encoding for putative bacteriocins in the genome of *Lc. paracasei* OLXI-9 were detected *in silico* using the BAGEL tool. These genes were annotated as Carnocin CP52 (40) and Thermophilin A (41), produced by *Carnobacterium piscicola* and *Streptococcus thermophilus,* respectively, and were reported to inhibit *Listeria* and *Enterococcus*. However, in the present study, using the well diffusion method with several different strains of these bacteria, zones of inhibition were not observed, indicating that bacteriocins were not expressed under these experimental conditions.

### Evaluation of β-glucosidase activity

The β-glucosidase activity of *Lc. paracasei* OLXI-9 was determined, using *Lc. rhamnosus* OXLAL-2 as a positive control (Table S4). Both strains expressed β-glucosidase activity, consistent with the *in silico* predictions by BAGEL. However, activity from OLXI-9 was about 25% lower compared to OXLAL-2.

## DISCUSSION

In this study, three strains of *Lc. paracasei* were isolated from Greek fermented table olives, and their genome sequences were determined by whole-genome sequencing. Recent genomic studies have shown that the subspecies division of *L. paracasei* subsp. *paracasei* and *L. paracasei* subsp. *tolerans* may not fully reflect their phylogenetic and ecological diversity, suggesting the presence of multiple distinct genomic clades with a strong influence of the strain’s ecological niche (15). Given this diversity and the wide range of ecological sources—ranging from dairy products to plant-based fermentations and host-associated environments—we propose a refined classification scheme that includes three major phylogenetic clades and an ecological categorization according to origin (dairy vs non-dairy food and beverage systems) (Fig. 1). Our data demonstrate that olive-derived strains form a distinct subcluster within clade 3. This represents an ecological specialization that has not been previously reported.

The need for a more detailed classification of *Lc. paracasei* has been previously reported (42), and comparative genomic studies further highlight the extensive intraspecies variability even among isolates from similar niches. Moreover, analyses of gene content have demonstrated that dairy and non-dairy isolates display specific genomic adaptations to their respective environments (15). Our findings are consistent with this concept, as the olive-derived isolates represent a non-dairy, plant-associated lineage within the broader *Lc. paracasei* species. Therefore, the present study extends the growing body of evidence that supports a new genomic and ecological framework for the taxonomy of *Lc. paracasei*, consistent with its evolutionary adaptation and biotechnological potential, by revealing a distinct ecological lineage within clade 3.

In addition, strains OLXI-1 and OLXI-6 clustered together, while strain OLXI-9 formed a separate sub-clade, despite all three being isolated from Greek fermented table olives. This phylogenetic distinction may reflect unique genetic traits and niche-specific adaptations that differentiate each isolate. Ultimately, the nomadic lifestyle of the species can be inferred based on the phylogenetic tree and the wide variety of habitats in which they are found, from invertebrate hosts to raw or fermented vegetables and dairy products (43). In this context, the genomic profiles of OLXI-1, OLXI-9, and OLXI-6 support their classification among the nomadic lactobacilli. Their ability to thrive in diverse environments is further reflected in specific genomic features that may contribute to environmental adaptability. For example, OLXI-1 contains approximately eight times more rRNA operons than the other two isolates (Table 1). Variation in rRNA copy number is known to influence microbial physiology because rRNA abundance controls the maximum rate of ribosome synthesis and, consequently, the potential for rapid protein production during periods of nutrient availability. In several bacterial groups, higher rRNA operon numbers are associated with faster growth responses and the ability to exploit transient resource pulses rather than being tied to genome size (44, 45). Although such relationships have not been extensively studied in *Lacticaseibacillus*, comparative work in lactic acid bacteria and related Firmicutes suggests that rRNA operon expansion can be driven by ecological pressures that favor rapid metabolic shifts (46, 47). Therefore, while direct evidence in *Lacticaseibacillus* is currently limited, the unusually high rRNA operon count in OLXI-1 may reflect an adaptive strategy that enhances responsiveness to fluctuating environmental conditions. This interpretation is consistent with broader patterns described for high-copy rRNA bacteria but should be considered a hypothesis requiring further investigation.

Regarding the CAZymes analysis, the three isolates showed differences in enzyme families within clade 3 (Fig. 2), suggesting an ability to adapt to diverse selective conditions (48). Beyond these general observations, we also examined additional CAZyme families relevant to host glycan degradation and prebiotic metabolism. The PL12 family enzymes degrade glycosaminoglycans (GAGs) and have been characterized structurally and enzymatically as heparinase III from *Bacteroides thetaiotaomicron* (49). GH88 enzymes are unsaturated glucuronyl hydrolases that also cleave sulfated GAGs, gellan, and xanthan (50, 51). GH136 family contains lacto-N-biosidase, a key enzyme that degrades lacto-N-tetraose, one of the main components of human milk oligosaccharides (HMOs), which is used by *Bifidobacterium longum* to metabolize HMOs (52). The CBM32 family, despite being very diverse, demonstrates binding specificity to galactose and lactose, which are commonly found in host glycans, such as GAGs, HMOs, and mucins (53). GT32 enzymes are involved in bacterial exopolysaccharide synthesis or O-glycosylation of host proteins.

Among the clade 3 genomes, the distribution of several CAZyme families is sporadic. GT14, GT101, and GH2 are present in OLXI-9 but are missing in OLXI-6 and OLXI-1. GT14 plays an important role in the biosynthesis of plant cell walls (54) but has never been characterized in bacteria. GT101 contains β-glucosyltransferases that have been previously reported in *Streptococcus parasanguinis* for O-glycosylation in bacterial adhesins (55). Families involved in FOS metabolism were also considered. GH32, present in most clade 3 genomes, including all three isolates, encodes invertases, levanases, inulinases, and levansucrases, all of which can generate fructo-conjugates (56, 57).

Members of the GH32 family exhibit substrate affinity toward levan, inulin, phlein, and various short-chain fructooligosaccharides, enabling the hydrolysis of β-fructosyl linkages characteristic of these fructans (58). Through this activity, GH32 enzymes release fructose or smaller fructo-oligosaccharides that can be readily imported and metabolized, providing a direct mechanistic explanation for the ability of our isolates to grow on inulin and FOS. The consistent association between the presence of GH32 genes and the observed fructan-utilization phenotypes across clade 3 genomes further underscores the functional importance of this CAZyme family in enabling efficient inulin metabolism.

In a study of 181 *Lc. paracasei* genomes (15), it was suggested that only about half could grow on inulin, highlighting the biological relevance of the strong fructan-utilization phenotype observed in our isolates. Although GH23 is present in our isolates and absent from many other clade 3 genomes, GH23 enzymes are generally associated with peptidoglycan remodeling (e.g., lytic transglycosylase/lysozyme activity) rather than fructan degradation (50). Thus, GH23 is unlikely to be the primary driver of inulin metabolism. Instead, its enrichment may reflect niche-specific adaptations that indirectly support growth or competitiveness in environments where fructans are present. In contrast, the presence of GH32—functionally linked to the hydrolysis of inulin and FOS—provides a more direct genomic explanation for the enhanced inulin utilization observed. Notably, genomes originating from animal hosts and non-dairy foods tended to encode more complete fructan-utilization gene sets than milk-derived isolates, with our isolates ranking among the strongest in predicted inulin utilization. These ecological patterns suggest that strain origin may influence the distribution and retention of genes involved in fructan metabolism.

Taken together, isolate OLXI-9 stood out among clade 3 members for encoding a distinct repertoire of CAZymes, including GT14, GT101, GH2, GH23, and GH32, which are absent or only sporadically present in other genomes (Fig. 2). In our *in vitro* assays, OLXI-9 showed high utilization of the inulin and FOS, consistent with its CAZyme profile. This strain also displayed the highest GH32 gene counts among clade 3 genomes (Fig. 2), enabling robust growth with inulin as the main energy source. Furthermore, OLXI-9 was able to ferment carbohydrates from a commercial fiber-rich olive pomace powder, although this material also contained glucose and other monosaccharides. Still, olive pomace has previously been suggested to have prebiotic properties during *in vitro* and *ex vivo* fermentations (32–34).

Most strains in clade 3 do not have GT14 genes, but they were present in OLXI-9. Additionally, GT101 and GT14 were either both present or absent in the genomes of clade 3, which could reveal a common evolutionary path. Moreover, GT2 and GT4 are the most prevalent and abundant families and are found in ancient Archaea; the other GT families may have evolved from these two families (59).

In the *in vitro* studies, isolate OLXI-9 utilized the prebiotic substrates inulin and FOS, indicating this strain could be a good candidate for a synbiotic containing these fructans. In addition, OLXI-9 also grew well on an olive pomace powder that claims to have prebiotic properties, although this material may have contained simple sugars, and we did not determine which specific substrates in the commercial material were metabolized. Finally, the *in vitro* β-glucosidase activity was confirmed in OLXI-9. Interestingly, it has been previously reported that β-glucosidase enzyme is important for oleuropein hydrolysis, which is responsible for reducing bitterness in fermented table olives (7).

In conclusion, in this study, we showed that strains of *Lacticaseibacillus paracasei* isolated from Greek fermented table olives have genetic features suggestive of metabolic versatility, including the ability to utilize a wide range of carbohydrates and prebiotic fibers. Accordingly, these findings provide a basis for understanding how fermentation-associated microbes grow on prebiotics and dietary fibers and potentially contribute to human health.

By integrating phenotypic assays with CAZyme profiling and comparative genomics, we reveal that plant-/olive-associated strains harbor a unique carbohydrate-degrading repertoire indicative of adaptation to fructan-rich environments. These results advance our current understanding of *Lc. paracasei* population genomic structure, revealing that niche-specific selective pressures contribute to microevolutionary divergence within the species.

## Data Availability

*Lc. paracasei* OLXI-1, OLXI-9, and OLXI-6 are part of the collection of the iFUNfoods Project (project code: T1EDK-03846), which was funded by the European Regional Development Fund of the European Union and Greek national funds through the Operational Program Competitiveness, Entrepreneurship, and Innovation, under the call RESEARCH CREATE—INNOVATE. Sample information of the three strains is available at NCBI under PRJNA1370124. The nucleotide sequences have been deposited at GenBank under the accession numbers JBSYUL000000000–JBSYUN000000000. The protein sequences and genome annotations are available at https://doi.org/10.6084/m9.figshare.31018603.

## SUPPLEMENTAL MATERIAL

Table S1 aem.00515-26-s0001.xlsx Prophages in the three genomes.

Table S2 aem.00515-26-s0002.xlsx CGCs in the three genomes.

Table S3 aem.00515-26-s0003.xlsx Biochemical profile.

Table S4 aem.00515-26-s0004.xlsx Beta-glucosidase activity.

ASM does not own the copyrights to Supplemental Material that may be linked to, or accessed through, an article. The authors have granted ASM a non-exclusive, world-wide license to publish the Supplemental Material files. Please contact the corresponding author directly for reuse.
